# Culturally Adapted Lifestyle and Mental Health Intervention for Low-Income Pregnant Women: A Feasibility Study

**DOI:** 10.1177/01939459251403005

**Published:** 2025-12-29

**Authors:** Maria Arminda Nunes, Bernadette Melnyk, Sofia Almeida, Margarida Vieira, Alexandrina Cardoso

**Affiliations:** 1 Institute of Health Sciences, Centre for Interdisciplinary Research in Health, Universidade Católica Portuguesa, Porto, Portugal; 2 Escola Superior de Enfermagem da Universidade do Porto, Portugal; 3 The Ohio State University, Columbus, OH, USA

**Keywords:** healthy lifestyle, mental health, pregnancy, low-income, culturally adapted intervention, feasibility study

## Abstract

**Background:**

Low-income pregnant women face challenges in maintaining a healthy lifestyle during pregnancy and protecting their mental health, increasing their risk of adverse perinatal outcomes. The Creating Opportunities for Personal Empowerment (COPE) program, culturally adapted for the Portuguese context, aims to promotes a healthy lifestyle and mental health.

**Objective:**

We aimed to assess the feasibility, acceptability, and preliminary effects of the culturally adapted intervention among low-income pregnant women, comparing in-person and online modalities.

**Methods:**

This mixed-methods study followed the Medical Research Council framework. Phase I involved cultural and linguistic adaptation of COPE using the ADAPT model. Phase II was a pre-post feasibility study with 45 low-income pregnant women attending in-person or online sessions. Feasibility was assessed through recruitment and retention. Acceptability was evaluated via engagement in skill-building activities, session rescheduling, and qualitative feedback. Preliminary effects were measured at T0 (baseline), T1 (post-intervention), and T2 (4-6 weeks postpartum).

**Results:**

Recruitment was 65.2%, with 68.9% retention, higher in the online group. On average, participants completed 3 skill-building activities and rescheduled 2.3 sessions. Qualitative feedback supported the intervention’s acceptability. Anxiety and depressive symptoms significantly decreased from T0 to T1, with anxiety reduction sustained at T2. Postpartum depression declined in the in-person group but increased online. Perceived stress remained unchanged, while healthy lifestyle beliefs and behaviors improved significantly.

**Conclusion:**

The COPE intervention was feasible and acceptable, demonstrating improvements in lifestyle behaviors and mental health. Its cultural adaptation supports applicability in the Portuguese context and highlights potential for broader international transfer.

**Trial Registration:**

Open Science Framework (https://doi.org/10.17605/OSF.IO/SQ5GK).

## Introduction

A healthy lifestyle during pregnancy is essential for maternal and fetal health, influencing perinatal outcomes and the child’s long-term development. Proper nutrition, physical activity, adequate sleep, and stress management reduce risks such as gestational diabetes, hypertension, and preterm birth.^1^ However, these practices are more difficult for low-income pregnant women, who face structural, financial, and social barriers that exacerbate health disparities.^2-4^

In Portugal, low-income disproportionately affects unemployed individuals, families with children, single-parent households, and those with lower educational levels.^5^ These groups are therefore considered socially vulnerable, as described in UNESCO’s Universal Declaration on Bioethics and Human Rights, which highlights how poverty, exclusion, and limited access to resources lead to social vulnerability.^6^ For pregnant women, these disadvantages often translate into limited access to nutritious food, greater reliance on energy-dense foods, and fewer opportunities for safe physical activity, contributing to excessive weight gain, gestational hypertension, and metabolic disturbances.^7-9^

Beyond lifestyle behaviors, insufficient income also has important consequences for mental health. Women experiencing financial insecurity are at increased risk of prenatal anxiety and depression, which are linked to adverse birth outcomes, including low birth weight, preterm delivery, and impaired mother-infant bonding.^10-12^ High stress levels are associated with elevated cortisol, which may disrupt fetal development and increase the likelihood of long-term neurodevelopmental challenges in offspring.^13,14^ Furthermore, maternal distress often leads to maladaptive coping behaviors such as overeating, smoking and alcohol consumption, exacerbating risks for both the mother and the child.^15,16^

Despite these challenges, pregnancy represents an opportunity for behavioral change, as women tend to be highly motivated to adopt healthier lifestyles for the well-being of their unborn child.^17,18^ However, in Portugal, there is a lack of structured, evidence-based interventions specifically designed to support low-income pregnant women in maintaining a healthy lifestyle. Culturally adapted interventions are needed to ensure alignment with local healthcare structures and population needs.

### Adapting Evidence-Based Interventions to Address Maternal Health Disparities

Addressing these multiple determinants requires structured, accessible, and sustained support within antenatal care. As frontline healthcare providers, nurse-midwives play a critical role in integrating evidence-based health promotion strategies into routine care. By fostering maternal health literacy, supporting behavioral change, and providing emotional support, they can mitigate the impact of social vulnerabilities on maternal and neonatal outcomes.^19^

The Creating Opportunities for Personal Empowerment (COPE) intervention was originally developed in the United States as a cognitive-behavioral skill-building program designed to promote healthy lifestyle behaviors, emotional regulation, problem-solving, and goal-setting across different populations.^20^ Grounded in universal principles of health promotion (eg, healthy diet, regular physical activity, stress management), COPE is also flexible to accommodate cultural diversity, as the specific ways of adopting these behaviors remain the responsibility and choice of each individual, depending on her cultural context, resources, and preferences.^20^

COPE’s effectiveness has been demonstrated in diverse groups, including adolescents,^21^ adolescents with chronic headaches,^22^ children who are victims of bullying,^23^ parents of preschool children,^24^ and students, academic, and nonacademic staff in university.^25^ More recently, COPE has also been adapted and tested on vulnerable pregnant women in the United States, showing improvements in lifestyle beliefs and behaviors, as well as reductions in anxiety, stress, and depressive symptoms.^26^ Additionally, COPE has been linked to lower healthcare costs, highlighting its cost-effective as a strategy to improving mental health.^27,28^

Building on this versatility, COPE was selected for cultural adaptation to the Portuguese context. Adapting evidence-based interventions allows for alignment with local healthcare systems, sociocultural norms, and population needs while maintaining core intervention components.^29^ Compared to developing a new intervention, cultural adaptation accelerates implementation, optimizes resource use, and improves intervention acceptability and effectiveness.^30^

## Purpose

Following the cultural adaptation process, this study aimed to assess the feasibility, acceptability, and preliminary effects of the culturally adapted COPE intervention among low-income pregnant women in Portugal, comparing in-person and online modalities.

## Methods

This mixed-methods feasibility study followed the Medical Research Council (MRC) framework for complex interventions^30^ and was conducted in 2 phases.

### Phase I: Cultural Adaptation of COPE

The cultural adaptation process was based on the ADAPT model,^29^ which provides a structured approach for transferring evidence-based interventions across contexts.

1. *Comparative Analysis:* A narrative literature review was conducted to evaluate COPE’s theoretical framework, evidence of effectiveness in different populations, and modes of delivery (individual, group, in-person, or online). Additionally, we analyzed the similarities and differences between the United States and Portuguese healthcare systems, focusing on the structure of primary health care, access to antenatal services, and the role of nurse-midwives. This step aimed to assess the contextual fit of COPE and identify elements requiring adaptation for successful implementation in Portugal.
2. *Consultation With the COPE Team:* Regular meetings clarified the components of the intervention and guided adaptation decisions about contextual adjustments, recruitment, and delivery.
3. *Stakeholder Engagement:* One focus group (*n* = 27) was conducted with healthcare professionals from primary care settings to assess the feasibility of implementing COPE in Portugal. Participants included nurse-midwives, nurses, physicians, psychologists, and administrative staff involved in maternal healthcare. The discussion aimed to identify facilitators, barriers, and practical strategies for integrating the intervention into routine antenatal care. The 60-minute session was moderated by 1 researcher, with another taking detailed field notes to ensure accurate documentation. The information gathered was reviewed and summarized to inform cultural and contextual adaptation decisions.
4. *Translation and Cultural Adaptation:* The COPE manual was culturally adapted following a structured 4-step process to ensure semantic, idiomatic, and conceptual equivalence.^31^ First, the manual was translated into Portuguese by the principal investigator. Two bilingual experts independently reviewed the translation. Next, a panel of experts reviewed and refined the content to ensure its cultural relevance. Finally, the Portuguese version was discussed with the original COPE developers during session simulations to verify conceptual equivalence and preserve theoretical fidelity.
5. *Training Simulations:* The principal investigator completed COPE training with the original development team. This included simulated delivery of all 7 sessions using the Portuguese manual to ensure comprehension, fidelity to the theoretical model, and consistency in the delivery process prior to the feasibility phase.

### Phase II: Feasibility Study

#### Participants and Recruitment

Participants were recruited from primary healthcare units in northern Portugal (May 2023-March 2024) using convenience sampling. The inclusion criteria were as follows: (1) pregnancy of ≤20 weeks gestation, (2) fluency in Portuguese, and (3) low-income status, defined as eligibility for healthcare fee exemptions. The exclusion criterion was a diagnosed psychiatric illness, due to the potential bias it could introduce in measures of anxiety, stress, and depression. Eligible participants were recruited during antenatal visits and informed consent was obtained. The sample size was determined to align with recommendations for feasibility studies, ensuring sufficient data to assess recruitment, retention, and acceptability while identifying potential implementation challenges before a larger trial.^30^

#### Intervention

COPE was delivered between June 2023 and April 2024, consisting of 7 weekly 30-minute sessions based on cognitive behavioral therapy (CBT) principles. In the first 6 sessions, participants were assigned skill-building activities to integrate the content into their daily routines, while the seventh session served as a closing session. Sessions were conducted individually or in small groups (3-6 participants), according to participants’ availability. An overview of session content is provided in Table 1.

**Table 1.** Content of COPE Sessions.

Session	Content
1	Thinking, feeling, and behaving: What is the connection?
2	Self-esteem and positive thinking
3	Stress and coping
4	Problem solving and setting goals
5	Dealing with your emotions in healthy ways through positive thinking and effective communication
6	Coping with stressful situations and healthy sleep
7	Pulling it all together for a healthy YOU!

Abbreviation: COPE, Creating Opportunities for Personal Empowerment.

**Figure 1.**
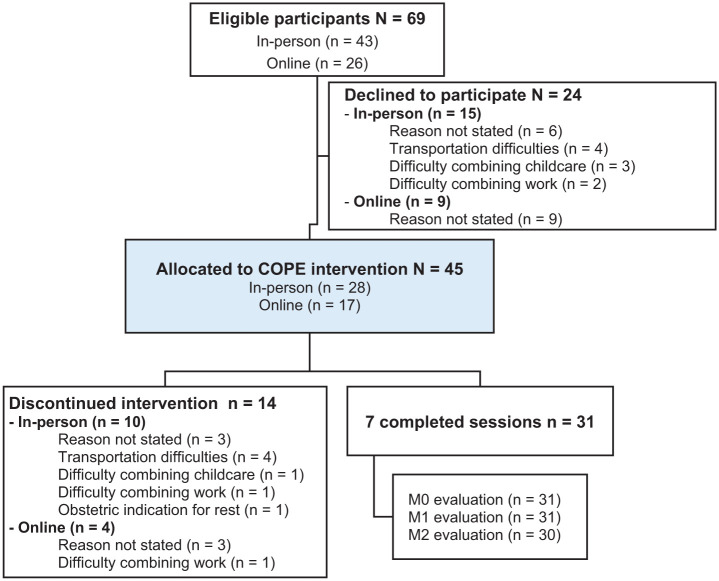
Flowchart of participant recruitment and retention.

From June to November 2023, the intervention was delivered in person (*n* = 28) at primary healthcare centers. Due to recruitment and retention difficulties (eg, transportation barriers, scheduling conflicts), from November 2023 to April 2024, sessions were delivered online via synchronous videoconferencing (*n* = 17; Figure 1). This responsive adaptation was in line with the MRC framework’s recommendations for flexibility in feasibility studies.^30^

All sessions were delivered by the same trained facilitator and scheduled according to each participant’s availability, including outside of regular working hours when necessary. To maximize adherence, sessions were rescheduled whenever possible. Participants who missed more than 2 consecutive sessions without rescheduling were considered to have discontinued the intervention.

#### Measures

Feasibility was assessed through evaluation of recruitment (the proportion of eligible participants who initiated the intervention), retention (the proportion of participants who completed all 7 sessions), and documented reasons for nonparticipation/discontinuation. Acceptability was evaluated via engagement in skill-building activities (mean number completed), session rescheduling, and qualitative feedback. Preliminary effects were assessed at T0 (baseline), T1 (post-intervention), and T2 (4-6 weeks postpartum) using validated scales:

- The Healthy Lifestyle Behaviors Scale (HLBES) evaluates self-reported healthy behaviors, scored from 0 to 60, where higher scores reflect healthier behaviors (Cronbach’s α = 0.78).^32^
- The Healthy Lifestyle Beliefs Scale (HLBS) assesses confidence in adopting healthy lifestyle, covering physical activity, nutrition, coping, and social support. Scores range from 0 to 75, with higher scores indicating stronger lifestyle beliefs (Cronbach’s α = 0.83).^33^
- The Generalized Anxiety Disorder-7 (GAD-7) Scale assesses anxiety levels, categorized as normal (0-4), low (5-9), moderate (10-14), or high (15-21; Cronbach’s α = 0.88).^34^
- The Perceived Stress Scale-10 (PSS-10) measures perceived stress on a scale from 0 to 40, with higher scores reflecting greater stress levels (Cronbach’s α = 0.87).^35^
- The Edinburgh Postnatal Depression Scale (EPDS) evaluates women’s depressive symptoms, with scores ranging from 0 to 30; scores ≥12 indicate moderate depressive symptoms (Cronbach’s α = 0.85).^36^

#### Data Analysis

Data analysis was conducted using IBM SPSS Statistics version 28.0 (IBM Corp, Armonk, NY, USA). Descriptive statistics were used to summarize participant characteristics and study variables. Categorical variables were described by relative frequency and absolute frequency, while continuous variables were described by mean and standard deviation.

To assess baseline comparability between intervention modalities (in-person vs online), chi-squared (χ^2^) tests were used for categorical variables, and Wilcoxon-Mann-Whitney tests (*U*) were applied for continuous variables, since normality assumptions were not met.

The effects of the intervention over time (T0, T1, T2) were analyzed using 1-way repeated measures ANOVA for the total sample, after confirming the assumptions of normality and sphericity. Mauchly’s test of sphericity was conducted, and if violated, Greenhouse-Geisser corrections were applied. To compare intervention effects between modalities (in-person vs online), a mixed repeated measures ANOVA was conducted. When significant main effects were found, Bonferroni post-hoc tests were conducted to determine specific differences between time points. The significance level was set at *P* < .05 for all analyses.

Qualitative data from open-ended questions were collected post-intervention (T1) to explore participants’ perceptions of acceptability. Questions addressed perceived usefulness of COPE, relevance of specific topics, behavioral changes, and suggestions for improvement, which were developed by the research team in alignment with previous studies adapting COPE to other populations.^23^

Responses were analyzed following Bardin’s content analysis methodology.^37^ The process was conducted manually in 3 phases: (1) pre-analysis, with repeated reading of the responses to become familiar with the material; (2) coding and categorization, where meaning units were manually highlighted and grouped into categories; and (3) interpretation, in which categories were synthesized into overarching themes reflecting women’s experiences. Two researchers independently coded the data, discussed discrepancies, and reached consensus.

### Ethical Considerations

The study was approved by the Northern Regional Health Administration Ethics Committee (protocol number: CE/2022/99), and all participants provided informed consent.

## Results

### Phase I: Cultural Adaptation of COPE

The adaptation process confirmed the feasibility of implementing COPE in Portuguese low-income pregnant women while maintaining fidelity to its CBT framework. The literature review reinforced COPE’s effectiveness in promoting healthy lifestyle beliefs and behaviors and improving emotional well-being in vulnerable populations, including pregnant women from minority backgrounds in the United States.^20,26,38,39^ The theoretical foundation of COPE aligned with maternal health interventions aimed at fostering self-efficacy, problem-solving, and emotional regulation, supporting its applicability in the Portuguese context.

Although the health systems in the United States and Portugal differ in their structure and financing, the implementation of COPE in antenatal care has basic similarities that are relevant to its transferability. In the United States, COPE was provided by nurse-midwives in community health centers that served low-income women without private health insurance. Similarly, in Portugal, antenatal care is universally accessible through the National Health Service, where nurse-midwives provide routine antenatal follow-up and health promotion in primary health care. Thus, in both contexts, women’s access to COPE would be free of charge and integrated into existing community maternal health services. In this analysis, the intervention was considered transferable, as the professional roles, target populations, and care delivery contexts were comparable. This approach prioritized the identification of elements that could influence transferability, rather than exhaustively cataloging all differences between the 2 contexts, in line with the guidelines of Moore et al,^29^ who emphasize that an excessive focus on contextual differences can lead to unnecessary modifications to the intervention.

Meetings with the original COPE developers helped clarify recruitment procedures, session structure, and skill-building activities. To address retention challenges, flexible scheduling and online delivery options were considered. Their input supported culturally appropriate adjustments while maintaining fidelity to the intervention’s CBT framework.

Healthcare professionals (*n* = 27) provided input regarding the feasibility of integrating COPE into routine antenatal care in Portugal. Nurse midwives emphasized the program’s alignment with their role in maternal health promotion, making them ideal facilitators. Stakeholders identified transportation barriers, scheduling conflicts, and the need for an online option as critical factors influencing participation. Their feedback informed engagement strategies and implementation logistics to enhance feasibility.

The Portuguese version of COPE maintained the original structure and theoretical framework while improving cultural sensitivity and comprehension. The main adaptations included adjusting the food examples to suit Portuguese eating habits, revising idiomatic expressions for clarity, and modifying visual elements to reflect culturally Portuguese contexts. These changes maintained the core content of the intervention and the therapeutic intention, while making it more sensitive to the culture of Portuguese pregnant women.

The simulated training sessions confirmed that the adapted Portuguese manual preserved the theoretical structure and therapeutic intent of the original COPE. The language adjustments improved clarity and accessibility for participants without compromising conceptual fidelity.

### Phase II: Feasibility Study

#### Participant Characteristics

A total of 45 participants were enrolled, with 28 in the in-person group and 17 in the online group. The mean age was 30.4 years (SD = 6.1), ranging from 18 to 41 years. Most participants had lower (46.7%) or secondary education (37.8%), and 57.8% were employed. Nearly all (97.8%) lived with the baby’s father, and households had an average of 3.9 members (SD = 1.7), ranging from 2 to 11. All participants were of Portuguese nationality. No significant differences were found between the in-person and online groups in any demographic variable, confirming baseline comparability (Table 2).

**Table 2.** Demographic Characteristics of Sample Group.

Variables	Total (*N* = 45)	In-person (*n* = 28)	Online (*n* = 17)	Test statistic	*P*
Age, *M ± SD*, y	30.4 ± 6.1	30.5 ± 6.4	30.0 ± 5.4	*U* = 232.0	.888
Nationality, *n* (%)					
Portuguese	45 (100)	28 (100)	17 (100)		
Education, *n* (%)				χ^2^ (2) = 4.960	.106
Lower education	21 (46.7)	10 (35.7)	11 (64.7)		
Secondary education	17 (37.8)	14 (50.0)	3 (17.6)		
Bachelor or higher	7 (15.6)	4 (14.3)	3 (17.6)		
Employment, *n* (%)				χ^2^ (1) = 0.178	.673
Employed	26 (57.8)	15 (53.6)	11 (64.7)		
Not employed	19 (42.2)	13 (46.4)	6 (35.3)		
Living with baby’s father, *n* (%)	44 (97.8)	28 (100)	16 (94.1)	χ^2^ (1) = 1.683	.194
Household members, *M ± SD*	3.9 ± 1.7	3.6 ± 0.9	4.5 ± 2.5	*U* = 198	.326

#### Feasibility Outcomes

Of the 69 eligible participants, 45 agreed to participate, yielding a 65.2% recruitment rate, consistent across both delivery modalities (65.1% in-person; 65.4% online). Retention was higher in the online modality (76.5%) than in the in-person modality (64.3%), with 31 participants (68.9% of total sample) completing all 7 sessions. Delivering COPE in groups proved challenging, since it was difficult to coordinate session times for 3 or more participants who met the inclusion criteria. Consequently, only 9 pregnant women started in groups (3 groups of 3), and 4 completed all 7 sessions. All participants who attended the first 3 sessions completed the intervention. Dropout reasons primarily involved logistical barriers such as transportation, childcare, and work commitments (Figure 1).

#### Acceptability Outcomes

Participants completed an average of 3.0 (SD = 1.7) out of 6 expected skill-building activities, with no significant difference between in-person (*M* = 3.0, SD = 1.4) and online (*M* = 2.9, SD = 2.1) groups. The main barriers were time constraints and forgetfulness, though many participants reported applying COPE strategies without formal documentation. Engagement improved over time, with 70.9% (*n* = 22) completing activities in the final 2 sessions.

Session rescheduling was common, with an average of 2.3 (SD = 1.4) rescheduled sessions, slightly higher in the online group (*M* = 2.5, SD = 1.7) than in-person (*M* = 2.1, SD = 1.3). While 12.9% attended all sessions without any rescheduling, most cited work and family responsibilities as reasons for adjustments.

The content analysis identified 4 key categories reflecting participants’ experiences with COPE:

- *Perceived Usefulness:* Participants valued COPE for stress management, emotional regulation, and promoting healthy behaviors. Some highlighted its importance for specific health conditions (eg, diabetes). Example comments: “*It was very useful. It greatly improved how I see and handle daily and pregnancy-related situations that would have been a problem before”* (P10); “*Yes, it helps a lot in maintaining a healthy lifestyle”* (P13); “*It was important because I have diabetes”* (P20).
- *Process Indicators:* Many participants engaged with and integrated COPE strategies into their daily life. Example comments: “*Gratitude, talking with friends, meditation, and deep breathing”* (P3); “*Handling stress, maintaining positive thoughts, and being grateful”* (P10); “*Living in the moment, being grateful for things”* (P19); and “*I started doing macramé to focus”* (P35).
- *Behavioral and Emotional Changes:* Positive lifestyle modifications were reported, including increased physical activity and improved dietary habits. Emotional well-being also improved, with participants reporting reduced stress and improved mood regulation. Example comments are as follows: “*I started exercising, be careful with my weight”* (P8); “*Less soda, I started Pilates and walking”* (P11); *“I’d rather go for a walk in the park with my daughter than watch TV”* (P26); *“I am calmer”* (P3); “*Less stress and worry”* (P8); “*I get angry less often”* (P16).
- *Suggested Improvements:* Participants recommend extending the program duration, increasing digital accessibility, and incorporating more group interactions. Example comments are as follows: “*Keep the program until nine months”* (P3); “*Repeat it after birth”* (P6); “*Using WhatsApp could help”* (P19); “*It would be interesting in a group setting”* (P22).

#### Preliminary Effects

The COPE intervention led to improvements in lifestyle behaviors, beliefs, and mental health outcomes over time in the total sample (Table 3). HLBES scores increased significantly (*F*_2,58_ = 32.468; *P* < .001), as did scores on the HLBS (*F*_2,58_ = 18.783; *P* < .001). GAD-7 scores declined (*F*_2,58_ = 13.347; *P* < .001), and EPDS scores also decreased (*F*_1,29_ = 4.525; *P* = .042). In contrast, PSS-10 scores did not change significantly over time (*F*_2,58_ = 1.514; *P* = .228).

**Table 3.** Effect of the COPE Intervention on the Total Sample (*N* = 30).

Measure	T0	T1	T2	One-way ANOVA	*P*
	*M* ± SD	*M* ± SD	*M* ± SD		
HLBES	41.0 ± 6.4	48.0 ± 5.4	48.4 ± 5.0	*F*_2,58_ = 32.468	.001
HLBS	53.7 ± 7.5	61.5 ± 5.2	62.3 ± 6.0	*F*_2,58_ = 18.783	.001
GAD-7	5.4 ± 2.1	3.2 ± 2.4	3.3 ± 2.6	*F*_2,58_ = 13.347	.001
PSS-10	13.5 ± 4.6	11.7 ± 4.8	11.8 ± 5.6	*F*_2,58_ = 1.514	.228
EPDS	6.2 ± 2.8	4.3 ± 2.6	5.3 ± 3.0	*F*_1,29_ = 4.525	.042

Abbreviations: COPE, Creating Opportunities for Personal Empowerment; EPDS, Edinburgh Postnatal Depression Scale; GAD-7, Generalized Anxiety Disorder-7; HLBES, Healthy Lifestyle Behaviors Scale; HLBS, Healthy Lifestyle Beliefs Scale; PSS-10, Perceived Stress Scale-10.

Table 4 compares outcomes between in-person and online modalities. For the HLBES, scores increased significantly over time (*F*_2,56_ = 30.905; *P* < .001), with post hoc tests confirming increases from T0 to T1 (95% CI, 4.69-9.61; *P* < .001) and from T0 to T2 (95% CI, 4.13-10.90; *P* < .001). HLBS scores followed the same pattern, *F*_2,56_ = 16.36; *P* < .001, with increases from T0 to T1 (95% CI, 3.07-11.23; *P* < .001) and T0 to T2 (95% CI, 3.42-11.91; *P* < .001).

**Table 4.** Comparison of Results Between In-Person and Online Modalities (*N* = 30).

Measure	Group	T0 (*M* ± SD)	T1 (*M* ± SD)	T2 (*M* ± SD)	Group effect			Time effect			Interaction effect		
					*F*	df	*P*	*F*	df	*P*	*F*	df	*P*
HLBES	In-person	40.7 ± 7.3	49.8 ± 4.4	48.2 ± 4.4	0.184	1, 28	.671	30.905	2, 56	<.001	2.155	2, 56	.125
	Online	41.3 ± 5.2	46.5 ± 4.9	48.8 ± 6.0									
HLBS	In-person	52.7 ± 8.6	62.9 ± 5.2	63.7 ± 6.6	0.521	1, 28	.476	16.360	2, 56	<.001	3.058	2, 56	.055
	Online	55.9 ± 5.1	60.0 ± 4.2	60.3 ± 4.4									
GAD-7	In-person	5.2 ± 2.6	3.6 ± 2.8	3.2 ± 3.0	0.131	1, 28	.721	14.806	2, 54	<.001	2.177	2, 54	.123
	Online	5.6 ± 1.4	2.2 ± 0.9	3.5 ± 2.1									
PSS-10	In-person	12.4 ± 5.2	11.8 ± 5.7	11.1 ± 6.6	0.539	1, 28	.469	1.797	2, 56	.175	0.971	2, 56	.385
	Online	14.3 ± 2.8	11.1 ± 2.7	12.9 ± 3.6									
EPDS	In-person	6.3 ± 2.2	4.5 ± 2.4	3.9 ± 2.3	0.674	1, 28	.419	6.248	2, 56	.004	8.401	2, 56	.001
	Online	5.6 ± 3.4	3.5 ± 1.8	7.3 ± 2.8									

Abbreviations: EPDS, Edinburgh Postnatal Depression Scale; GAD-7, Generalized Anxiety Disorder-7; HLBES, Healthy Lifestyle Behaviors Scale; HLBS, Healthy Lifestyle Beliefs Scale; PSS-10, Perceived Stress Scale-10.

For the GAD-7, scores decreased significantly over time (*F*_2,54_ = 14.806; *P* < .001), with post hoc analyses confirming reductions from T0 and T1 (95% CI, 1.38-3.62; *P* < .001) and from T0 to T2 (95% CI, 0.87-3.15; *P* < .001). The PSS-10 showed no significant change across the 3 time points (*F*_2,56_ = 1.797; *P* = .175).

**Figure 2.**
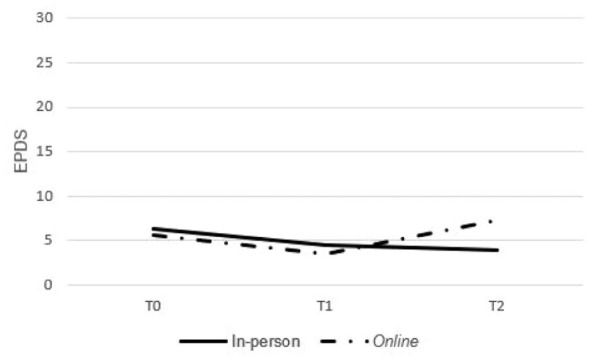
Evolution of the mean EPDS scores over time in in-person and online groups. EPDS, Edinburgh Postnatal Depression Scale.

For the EPDS, scores declined significantly over time (*F*_2,56_ = 6.248; *P* = .004), with a post hoc analysis confirming reductions from T0 to T1 (95% CI, 0.77-3.15; *P* < .001). A significant time-by-group interaction effect (*F*_2,56_ = 8.401; *P* = .001) indicated a sustained reduction in the in-person group, while scores in the online group increased again at T2 (Figure 2). No other significant group or interaction effects were observed (Table 4). Statistical comparisons between the group and individual formats were not feasible due to the limited number of participants who completed the group intervention (*n* = 4).

## Discussion

The COPE intervention was found to be feasible and acceptable for low-income pregnant women, with recruitment and retention rates comparable to similar interventions targeting vulnerable populations.^26,40^ Nonetheless, logistical barriers, such as transportation difficulties and competing family and work responsibilities, remained important challenges, consistent with previous maternal health intervention studies.^39,41^ The absence of explanations from many women who declined or discontinued participation limits interpretation. However, the literature indicates that nonparticipation in vulnerable populations is often linked to financial strain, limited social support, or difficulty committing to multi-session programs, while lack of familiarity with COPE as a new intervention may also have played a role.^42-45^ The slightly higher retention rate in the online modality suggests that digital interventions may help mitigate logistical barriers, though future adaptations may benefit from hybrid models that combine the accessibility of online sessions with the relational support of face-to-face contact.^46,47^

Notably, all participants who attended at least the first 3 sessions completed the intervention, underscoring the importance of early engagement as a key factor in promoting retention. This finding aligns with Meleis’ Transition Theory, which conceptualizes pregnancy as a developmental transition in which early involvement and support are critical for adaptation.^44^

Although adherence to the skills development activities was moderate, participants’ involvement increased over time, indicating a progressive integration of the intervention into their daily lives. Many women reported using COPE strategies, such as stress management techniques, relaxation exercises, and positive thinking, which may have strengthened their cognitive beliefs about their ability to engage in healthy lifestyle behaviors. These changes align with the observed improvements in lifestyle beliefs and behaviors measured in this study. The frequent need to reschedule sessions further illustrates the importance of flexible and adaptive approaches in implementation.^48,49^ Rather than being a limitation, this flexibility was incorporated into the cultural adaptation of COPE in Portugal, allowing the intervention to respond to participants’ daily constraints and thus reinforcing feasibility and acceptability.

COPE was associated with improvements in healthy lifestyle beliefs and behaviors, as well as mental health outcomes, among low-income pregnant women. The increase in HLBES and HLBS scores over time suggests that the intervention reinforced confidence in adopting and sustaining healthy behaviors, aligning with prior studies demonstrating COPE’s effectiveness in promoting lifestyle changes.^23,26^ These findings underscore the intervention’s potential to address health disparities by providing structured support to populations facing socioeconomic barriers.

Regarding mental health outcomes, COPE contributed to a significant reduction in anxiety and depressive symptoms. However, while anxiety reductions were sustained postpartum, depressive symptoms increased in the online group, resulting in a significant time-by-group interaction effect. This disparity may be linked to the absence of in-person contact in the online modality, underscoring the need for hybrid models that combine online accessibility with face-to-face sessions and, when necessary, home visits to overcome logistical barriers.^50^ Perceived stress levels remained unchanged throughout the intervention, suggesting that stress among low-income pregnant women may be shaped by broader structural and contextual determinants, such as financial strain and social support deficits, which require more prolonged or multidimensional interventions.^51^ This finding contrasts with a previous study,^26^ where perceived stress significantly decreased post-intervention, potentially indicating differences in sample characteristics or external support mechanisms.

A unique aspect of this study lies in the cultural adaptation of COPE to the Portuguese National Health Service, a universal and publicly funded system where nurse-midwives play a central role in antenatal care.^52^ This contrasts with previous implementations in the United States, where COPE was delivered in community health centers primarily serving low-income pregnant women without private health insurance. In Portugal, because antenatal care is universally accessible, the cultural adaptation focused less on structural access to health care and more on addressing contextual and logistical challenges, such as transportation, scheduling, and competing family responsibilities. These barriers were addressed through responsive delivery strategies, including flexible hours and online modalities. More broadly, this adaptation demonstrates that evidence-based interventions like COPE can be successfully transferred across health systems with different structures, provided that implementation is grounded in shared professional roles and responsive to social determinants of health. This experience highlights key principles, such as stakeholder involvement, contextual tailoring, and preservation of core components, that can guide future COPE adaptations in other cultural and healthcare settings.^39,50,53^

These results also offer important implications for clinical practice, public health, and future research, highlighting the potential of COPE as a structured intervention for promoting healthy lifestyle behaviors and mental health among low-income pregnant women. The feasibility and acceptability findings emphasize the need for flexible and accessible interventions that address the logistical and socioeconomic barriers faced by this population. Given the observed improvements in healthy lifestyle and mental health, integrating COPE into routine antenatal care, particularly through nurse-led programs, could enhance maternal health support while fostering continuity of care.

Future research should prioritize large-scale randomized controlled trials to evaluate the long-term effectiveness of COPE and its impact on maternal and neonatal health outcomes. Expanding the sample size and incorporating a control group will allow for a more robust assessment of intervention efficacy and sustainability. Additionally, optimizing COPE delivery, particularly in online settings, remains a critical area for improvement, as engagement and long-term adherence require further refinement.

Public health initiatives should consider scaling COPE within healthcare systems, tailoring it to diverse populations, and integrating it into national maternal health policies. Investing in structured, evidence-based interventions for vulnerable pregnant women can contribute to better pregnancy outcomes, reduced healthcare costs, and long-term benefits for maternal and child health.^20,54,55^

### Limitations

This study has several limitations that should be considered when interpreting the findings. First, the self-reported nature of the data may have introduced a social desirability bias, particularly for lifestyle behaviors and emotional well-being, potentially leading to an overestimation of intervention effects. Second, the relatively small sample size, while appropriate for a feasibility study, limits the generalizability of findings and the statistical power to detect differences between delivery modalities. Participants were also not able to choose their preferred modality, which could have affected both engagement and outcomes, especially in relation to personal preferences, digital literacy, or comfort with technology. Third, the follow-up period was limited to pregnancy and the first-month postpartum, restricting the ability to assess the long-term sustainability of behavioral and emotional outcomes. Fourth, most participants lived with a partner, reducing generalizability to single mothers, who may face distinct social and economic challenges during pregnancy and postpartum. Finally, the sample consisted exclusively of persons of Portuguese nationality, which restricts the applicability of the findings to immigrant populations and more culturally diverse groups.

## Conclusion

This study provides preliminary evidence of the feasibility and acceptability of the culturally adapted COPE intervention for low-income pregnant women, with both in-person and online modalities demonstrating positive effects on healthy lifestyle beliefs and behaviors and mental health. Participants reported increased self-efficacy in adopting and maintaining healthy behaviors, and significant reductions in anxiety and depressive symptoms were observed following the intervention. However, while the reduction in anxiety persisted postpartum, depressive symptoms increased in the online group, suggesting that in-person interactions may provide additional emotional support. As an initial study, these findings support the progression to a large-scale evaluation of the efficacy of the adapted COPE intervention within the Portuguese context.
